# Requirements for benefit assessment in Germany and England – overview and comparison

**DOI:** 10.1186/s13561-014-0012-8

**Published:** 2014-08-28

**Authors:** Victor Ivandic

**Affiliations:** Promed writing, Grillparzerstr. 7, Freiburg, 79102 Germany; Center for Cognitive Science, University of Freiburg, Freiburg, 79102 Germany

**Keywords:** AMNOG, G-BA, Health technology appraisal (HTA), IQWiG, NICE

## Abstract

**Background:**

This study compared the methodological requirements for early health technology appraisal (HTA) by the Federal Joint Committee/Institute for Quality and Efficiency in Health Care (G-BA/IQWiG; Germany) and the National Institute for Health and Care Excellence (NICE; England).

**Methods:**

The following aspects were examined: guidance texts on methodology and information sources for the assessment; clinical study design and methodology; statistical analysis, quality of evidence base, extrapolation of results (modeling), and generalisability of study results; and categorisation of outcome.

**Results:**

There is some degree of similarity regarding basic methodological elements such as selection of information sources (e.g. preference of randomised controlled studies, RCTs) and quality assessment of the available evidence. Generally, the approach taken by NICE seems to be more open and less restrictive as compared with G-BA/IQWiG. Any kind of potentially relevant evidence is requested, including data from non-RCTs. Surrogate endpoints are also accepted more readily, if they are reasonably likely to predict clinical benefit. Modeling is expected to be performed wherever possible and appropriate, e.g. for study duration, patient population, choice of comparator, and type of outcomes. The resulting uncertainty is quantified through sensitivity analyses before making a recommendation regarding reimbursement. By contrast, G-BA/IQWiG bases its assessment and quantification of the additional benefit largely, if not exclusively, on evidence of the highest level and quality and on measurements of “hard” clinical endpoints. This more conservative approach rather firmly dismisses evidence from non-RCTs and measurements of surrogate endpoints that have not or only partly been validated. Moreover, neither qualitative extrapolation nor quantitative modeling of data is done.

**Conclusions:**

Methodological requirements differed mainly in the acceptance of low-level evidence, surrogate endpoints, and data modeling. Some of the discrepancies may be explained, at least in part, by differences in the health care system and procedural aspects (e.g. timing of assessment).

**Electronic supplementary material:**

The online version of this article (doi:10.1186/s13561-014-0012-8) contains supplementary material, which is available to authorized users.

## Background

In most Western countries, a comparative analysis of clinical efficacy and/or effectiveness is performed to support national decisions regarding reimbursement or pricing of a pharmaceutical drug or other health care technology. Benefit evaluations can be divided into rapid (or early) assessments for single new pharmaceuticals and full assessments for (all) available therapeutic options. For rapid assessments, which are usually performed soon after marketing authorisation, a timeframe is often pre-specified, e.g. 6 months for early benefit assessments in Germany and approx. 39 weeks for rapid health technology appraisal (HTA) in England. For full (non-rapid) assessments, which are often conducted when the pharmaceutical has already been available on the market for several years, there is usually no fixed timeframe (in England, full assessments are subject to a specific timeframe, approx. 54 weeks).

The conditions for acceptance of evidence for clinical benefit vary among national HTA agencies since the structure of the health care systems differs greatly from country to country. While the healthcare system in England, for example, is a primarily tax-based national health service, an insurance-based health system is in place in Germany (social or private health insurance) [1],[2]. In England, company profit rather than drug prices are subject to direct regulation, at present. Until end of 2010, drug prices in Germany were not regulated but were indirectly influenced by the reimbursement system (e.g. fixed pricing, agreements on discounts).

Since 1 January 2011 all new patent-protected pharmaceuticals undergo a systematic assessment of the additional benefit upon market entry in Germany. The purpose of the rapid benefit assessment pursuant to § 35a Social Code Book V (SGB V) [3] is primarily to support pricing decisions according to § 130b SGB V. The overall aim of the new legislation is to counteract the disproportional increase in costs for pharmaceuticals that are not yet regulated by the fixed pricing system. At the same time, the availability of innovative pharmaceuticals to the patients is to be maintained at adequate costs for the health care system.

**Figure 1:**
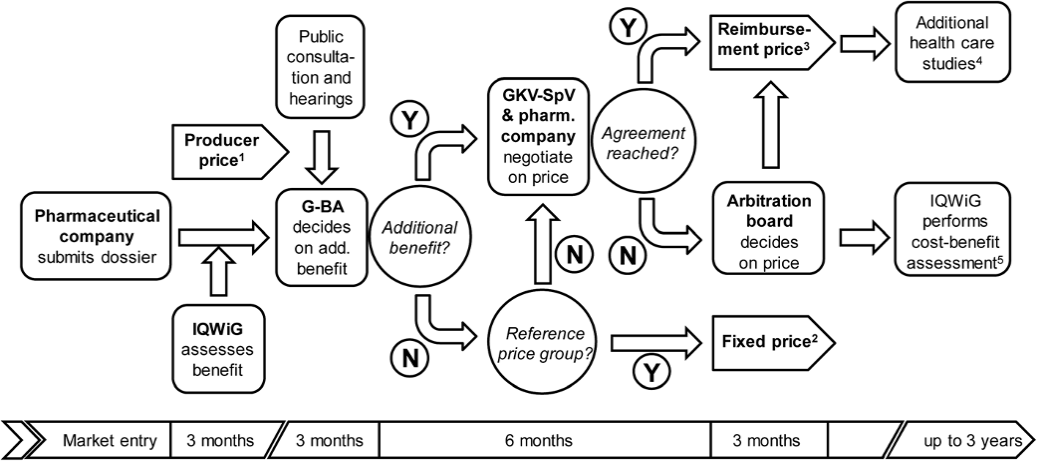
**Early benefit assessment in Germany according to § 35a SGB V (AMNOG).** Abbr.: AMNOG = German Law for Reforming the Market for Pharmaceuticals; G-BA = Federal Joint Committee; GKV-SpV = Central Federal Association of the Statutory Health Insurance Funds; IQWiG = Institute for Quality and Efficiency in Health Care; N = No; SGB V = Social Code Book V; Y = Yes. 1) effective for up to 1 year; 2) effective from end of 12-month assessment period; 3) with retroactive effect from end of 12-month assessment period; 4) if deemed required due to insufficient evidence base; 5) according to § 35b SGB V, if arbitrament is not accepted.

There are two HTA organisations that give input for reimbursement decisions of pharmaceuticals in Germany. The Institute for Quality and Efficiency in Health Care (IQWiG) usually carries out the benefit assessment and prepares a recommendation with a quantification of the additional benefit. The Federal Joint Committee (G-BA) makes the final decision regarding the benefit assessment. Subsequently, pharmaceutical company and Central Federal Association of the Statutory Health Insurance (GKV-SpV) agree on a reimbursement price (if fixed pricing is not possible), which takes into account the costs of the comparator therapy and the additional benefit of the new drug. If price negotiations fail, the price is set by a board of arbitration (Figure 1). A major difference between Germany and England is that in Germany drugs are reimbursed immediately after regulatory approval (Figure 1) and irrespective of the outcome of the early benefit assessment while in England reimbursement depends on the outcome of the early assessment (“fourth hurdle”).

The aim of the comparative benefit analysis in England is to provide input for national reimbursement decisions by the National Health Service (NHS). The technology appraisals are performed by the National Institute for Health and Care Excellence (NICE). As stipulated in the Directions of the Secretary of State [4], the purpose of the appraisal by NICE is to assess the clinical benefits and the costs of interventions and to make recommendations as to “whether, on balance, the intervention can be recommended as a cost-effective use of NHS resources in general, or for specific indications, or for defined subgroups of patients” [5]. Thus, clinical effectiveness and cost-effectiveness of a technology are examined in one and the same appraisal.

The outcome of HTA for a new medical technology in England is issued early, in form of guidance by NICE, and may thus be considered by HTA authorities in other EU countries. At the same time, England represents a major market within the EU for the pharmaceutical industry [6]. Therefore, in this study, the requirements for demonstrating the benefit of a drug in Germany were compared with those in England. The aim was to explore to which extent the requirements differ, a question of relevance e.g. to a pharmaceutical company wishing to submit a dossier for HTA in both countries. The findings are presented separately for the two HTA systems and thus may serve as stand-alone references. A concise, integrated comparison follows to highlight the main similarities and differences in the methodological requirements. Finally, possible explanations for discrepancies are explored.

## Methods

An overview is primarily given of the methodological requirements currently used for the relative effectiveness assessment by NICE in England and the early benefit assessment by G-BA/IQWiG in German. To allow comparison between early benefit assessment in Germany and single technology appraisal in England, the following aspects were examined: guidance texts on methodology and information sources for the assessment (e.g. dossier); clinical study design and methodology (e.g. study type and duration, comparator, endpoints); statistical analysis (e.g. systematic reviews, meta-analyses, sensitivity analyses), quality of evidence base, extrapolation of results (modeling), and generalisability of study results (i.e. external validity); and categorisation of outcome. The cut-off date for information considered for inclusion in the manuscript is 31 August 2013.

This study focused on the comparative evaluation of clinical effectiveness, which is always performed in both legal systems directly after market access (“early assessment”). The area of cost-effectiveness assessment, however, was not subject of this study as it does not form part of the initial, early benefit assessment in Germany. This step may be performed at a later stage under specific circumstances, e.g. if the parties have not come to an agreement during the regular price negotiation (Figure 1). By contrast, cost-effectiveness is always evaluated in England due to the nature and purpose of the appraisal; criteria for assessments include direct and indirect costs, e.g. social productivity; resource management; resource use identification, measurement, and costing; discounting; equity; and impact on NHS budget and services [5]. Regulatory and process-related aspects are presented where necessary, i.e. if relevant in the context of the evaluation or associated with methodological requirements. A detailed comparison of the legal or procedural elements is outside of the scope of the present review. Finally, the study is restricted to pharmaceutical drugs since the benefit assessment in Germany according to AMNOG does not apply to all types of health technologies.

## Results

### Methodological requirements for early benefit assessment in Germany (G-BA/IQWiG)

#### Legal basis, **guidance texts, and information sources**

##### Regulatory and guidance texts relevant to early benefit assessment

**Figure 2:**
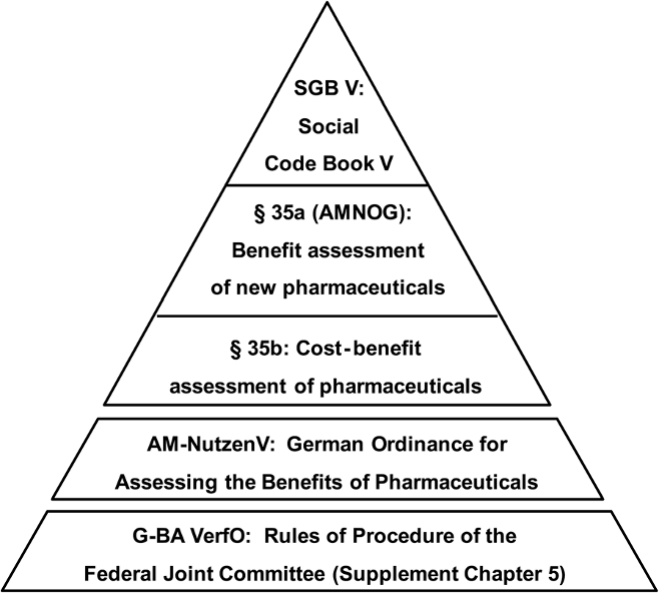
**Hierarchy of legal texts related to early benefit assessment in Germany.** Abbr. AMNOG = Arzneimittelmarkt-Neuordnungsgesetz (German Law for Reforming the Market for Pharmaceuticals).

The hierarchy of legal texts related to (cost-)benefit assessment is shown in Figure 2. The SGB V [3] is the legal basis of the statutory health insurance funds (GKV) in Germany. The German law for reforming the market for pharmaceuticals (AMNOG) introduced an entirely new means of price regulation for recently authorised pharmaceuticals by establishing a new § 35a SGB V on the “Assessment of benefit for pharmaceuticals with new active substances”. This early benefit assessment is mandatory for all new pharmaceuticals or new therapeutic indications authorised in Germany and is based on information provided by the pharmaceutical company (in form of a dossier).

The benefit assessment is described in greater detail in the German Ordinance for Assessing the Benefits of Pharmaceuticals (AM-NutzenV) [7]. This regulation entered into force as from 1 January 2011 - like the AMNOG - and provided the basis for the new supplement of G-BA’s Rules of Procedure.

The Rules of Procedure of G-BA (G-BA VerfO) are important as they specify the methodological basis for decision-making in many areas. In a resolution, which entered into force on 22 January 2011, G-BA added an additional chapter to its rules of procedure, which is entitled “Chapter 5: Assessment of the benefits of pharmaceuticals according to § 35a SGB V”. This supplement completes the AMNOG and related legal regulations by joining the regulations in an integrative set of rules. In Annex II of the Supplement of the G-BA VerfO (Suppl. G-BA VerfO) [8], the specifications on the format and structure of the dossiers and documents to be submitted are described in detail, and comprehensive guidance on the methodological requirements is given.

The methodology used for benefit assessment by IQWiG is described in form of a methods paper called “General Methods 4.0” (IQWiG GM 4.0), which is available publicly and in an English translation [9]. The level of detail on the methodology is very high.

##### Information sources

**Figure 3:**
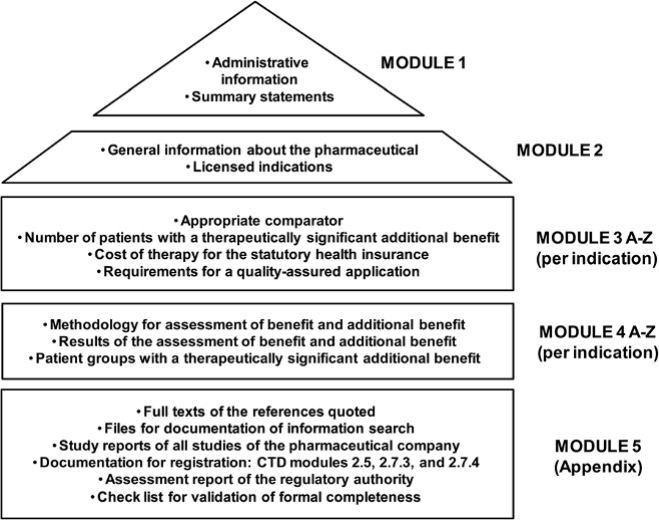
**Structure of the dossier for early benefit assessment in Germany.**

For an early benefit assessment according to § 35a SGB V, the main source is a dossier that is provided by the drug manufacturer. The structure and format of the dossier (Figure 3), the documents to be submitted and technical standards are pre-specified; mandatory and optional contents are described, examples and explanations are given, and placeholders are provided for text entries and tables to be filled with data. Finally, a check list is offered to test for formal completeness of the dossier. The size of the dossier (main body), which is written in German, usually exceeds 300 pages, and the volume of appendices is in the range of 1000s of pages.

For the first assessment, at time of marketing, the licensing studies form the basis of information in the dossier. In addition, a systematic literature search is always required (Section 4.2.3.2 Suppl. G-BA VerfO).

A literature search should be performed using the MEDLINE, EMBASE, and Cochrane databases as a minimum. Further details regarding the search strategies (e.g. cut-off date, block-wise search, or separate searches by indication, intervention and study type) are provided in Section 4.2.3.2 Suppl. G-BA VerfO. In any case, the process is to be documented, and deviations from the recommendations are to be justified.

The methodology used for systematic literature search should be suitable for identifying both published and unpublished studies (Section 4.2.3 Suppl. G-BA VerfO). To ensure that all on-going, prematurely discontinued and completed studies from third-party sponsors are identified, web-based study registries are always to be searched (Section 4.2.3.3 Suppl. G-BA VerfO).

Confidential data submitted in Module 5 of the dossier may also be part of the assessment. While content of Module 5 might be labeled as confidential, all information and data used in the assessment has to be part of Modules 1 to 4 and thus must be publicly available. Otherwise, proof of additional benefit is not considered to have been provided due to an incomplete dossier (§9 par. 3 G-BA VerfO; § 35a par. 1 SGB V).

During the dossier review by IQWiG, input from external medical experts and patients or patient organisation is sought through questionnaires (and by discussion) [10]. Furthermore, pharmaceutical companies, industry organisations, pharmacists’ associations, umbrella organisations of medical professionals, and scientific experts are given the opportunity (for 3 weeks) to make written comments on the assessment report, which is prepared by IQWiG and published by G-BA (§ 19 par. 1 s. 1 G-BA VerfO). Additionally, verbal hearings may be conducted after the written hearing procedure and before a final decision is made by G-BA (§ 35a par. 3 s. 2 SGB V in connection with § 19 par. 1 s. 2 G-BA VerfO).

As per § 5 par. 5 s. 2 AM-NutzenV, the available evidence is the basis for decision-making. Due to the early time point of benefit assessment, the evidence base may be limited. If the evidence provided is not deemed sufficient, e.g. because of poor result certainty or doubts regarding the safety or appropriateness, G-BA can demand additional evidence as part of its decision (§ 35b par. 2 SGB V). A deadline, by when these data should be provided (e.g. at the latest after 3 years) and the type of study design (e.g. pragmatic randomised controlled trials) are predetermined by G-BA (§ 92 par. 2a SGB V). The health care provision studies should preferably be conducted in Germany to ascertain that results are applicable to the local health care conditions.

#### Clinical study design and methodology

##### Study design features

The study design and methods of the included studies are to be described to ensure that the dossier contains all information relevant for benefit assessment (Section 4.2.5.1 Suppl. G-BA VerfO; Section 7.1.4 IQWiG GM 4.0). The requirements should at least comply with the established international standards for randomised studies (CONSORT) [11], non-randomised interventional studies (TREND-2) [12], and epidemiological observational studies (STROBE) [13]. The importance of study design aspects in benefit assessment (e.g. compliance to good clinical practice (GCP), sample size calculation, control group, blinding, randomisation, interim analyses, prospective and retrospective designs) is discussed in detail in Section 7.1.2 and 7.1.4 of the IQWiG GM 4.0.

Randomised clinical trials (RCTs) and, in particular, direct comparison trials are preferred to protect against selection bias in clinical studies through blinding and randomisation [14]. If it is not possible or inadequate to provide or generate best-level evidence, the pharmaceutical company may also submit best-available evidence, justifying its suitability for the benefit assessment (§ 5 par. 3 and 6 AM-NutzenV; § 5 par. 3 G-BA VerfO; Section 4.5.2 and 4.5.3 Suppl. G-BA VerfO). Finally, IQWiG may accept results from well documented case series, which have a lower validity, if the intervention brings about a “dramatic effect”, e.g. life-saving substitution of essential hormones (Section 3.2.2 IQWiG GM 4.0).

According to § 35a par. 1 SGB V, the number of patients and patient groups for which a therapeutically relevant additional benefit exists is to be determined as part of the benefit assessment. The extent of the additional benefit and its therapeutic importance are quantified using defined categories (§ 5 par. 7 AM-NutzenV) for each group of patients (§ 4 par. 1 and § 5 par. 4 AM-NutzenV). The results of subgroup analyses can help to identify these patients or patient groups (Section 4.2.5.5 Suppl. G-BA VerfO).

The G-BA VerfO (Section 4.2.5.5 Suppl. G-BA VerfO) further specifies that the effect modifiers sex, age, disease severity or disease stage as well as country and study centre-effects should always be analysed. In addition to these characteristics, other clinical factors related to the treatment (e.g. dose level) should be considered. Biometric tests to help avoid errors in interpretation of results are proposed (e.g. homogeneity or interaction tests).

According to IQWiG, subgroup analyses of other variables may, however, not always yield reliable results and are therefore viewed with great caution (Section 7.1.6 IQWiG GM 4.0), especially if i) if they are performed post-hoc, i.e. have not been planned *a priori* (as part of the study protocol or protocol amendments), ii) if numerous subgroups are analysed (multiple testing), and, hence, false positives may arise by chance (α-error), and iii) if the size of subgroups is small, i.e. if the power of the statistical analyses performed is low, and false negatives may arise by chance (β-error).

Study duration is considered an important criterion for identifying studies that are relevant for benefit assessment (Section 3.2.3 IQWiG GM 4.0). To determine the minimum study duration, indication-specific guidelines from regulatory authorities or international organisation (e.g. ICH) are taken into account.

##### Comparator and comparison

The benefit and additional benefit of a new pharmaceutical drug are assessed by comparison with a specific appropriate comparator (§ 2 par. 5 AM-NutzenV). The pharmaceutical company can choose from several comparators, which are determined by the G-BA (§ 6 par. 2a AM-NutzenV). The following criteria are taken into consideration: i) the comparator should be selected using methods that correspond to the international standards of evidence-based medicine (§ 6 par. 1 AM-NutzenV); ii) the comparator should be an appropriate therapy in the therapeutic indication according to the generally accepted state of medical knowledge (§ 6 par. 2 AM-NutzenV); iii) the comparator should preferably be a therapy for which there are endpoint studies and which has proven itself in practical use, unless this is in opposition to guidelines or the efficiency principle (§ 6 par. 2 AM-NutzenV); iv) if a pharmaceutical is considered as the comparator, the pharmaceutical must be authorised for the therapeutic indication (Section 3.1 Suppl. G-BA VerfO); v) if a non-medicinal treatment is considered as the comparator, this must be deliverable within the framework of the statutory health insurance (i.e. reimbursable) (Section 3.1 Suppl. G-BA VerfO); vi) those comparators are preferred whose patient-relevant benefit has already been determined (i.e. pre-assessed) by G-BA (Section 3.1 Suppl. G-BA VerfO); vii) for pharmaceuticals of an active substance class, the same appropriate comparator must be used to guarantee a uniform assessment (§ 6 par. 3 AM-NutzenV); viii) the comparator must also be suitable for assessing existing pharmaceuticals (upon request from G-BA) that were made available on the market before 1 January 2011 (Section 3.1 Suppl. G-BA VerfO).

Pharmaceutical companies can request consultation from G-BA on the choice of appropriate comparator, on design aspects of studies, and on the content of the documents to be submitted (§ 35a par. 7 SGB V). The consultation procedure may take place before phase 3 clinical studies or submission (§ 8 par. 1 AM-NutzenV).

While evidence from direct comparisons based on head-to-head RCTs is generally preferred, it may not be available or insufficient at the time of early benefit assessment (Section 7.3.9 IQWiG GM 4.0). Indirect comparisons may then be used to determine the relative effectiveness, provided studies are adequate for indirect comparisons (§ 5 par. 5 AM-NutzenV). Some methodological specifications are provided in the dossier template (Section 4.2.5.6 Suppl. G-BA VerfO). Non-adjusted indirect comparisons (i.e. comparisons based on studies without a common comparator) are considered inacceptable. Otherwise, no clear preference is given regarding the type of analysis used for comparing indirectly. Both simple [15] and complex adjusted analyses can be used, e.g. Bayesian analysis, mixed-treatment-comparison meta-analyses [16], multiple-treatment-meta-analyses [17] or network meta-analysis [18].

##### Clinical endpoints/outcomes

Patient-relevant outcomes are not only preferred, but required, as per definition of benefit in § 2 par. 3 AM-NutzenV; in particular mortality, morbidity (complaints and complications), and quality of life have to be taken into account. Only if patient-relevant endpoints cannot be made available, the assessment is based on other data (§ 5 par. 5 AM-NutzenV). Patient-reported outcomes such as health-related quality of life (HRQL) and symptom scores may also be used provided that the instruments used are validated (Section 3.2.4 IQWiG GM 4.0). As supplementary information, treatment satisfaction of patients may be taken into account (Section 3.1.1 IQWiG GM 4.0). Utilities such as quality-adjusted life years (QALYs) may also be considered in the assessment.

If data on patient-relevant endpoints are not available, surrogate endpoints may be used (Section 4.5.3 Suppl. G-BA VerfO). In general, only valid surrogate outcomes are accepted (Section 4.5.4 Suppl. G-BA VerfO). For validation, usually a meta-analysis is required, which investigates the effects of an intervention on both the surrogate outcome and the patient-relevant endpoint of interest [19],[20]. The studies included in the meta-analysis should be conducted in patient populations and using interventions, which are relevant to the indication, pharmaceutical and comparator to be assessed. The use of other alternative methods for validation e.g. [21] should be justified, especially if they are based on only one study. While it is accepted by IQWiG that surrogate endpoints are especially important for early benefit assessment, it is also emphasized that there is no validation standard, i.e. no ideal evaluation method or criterion which would generally be accepted to support validity of surrogate endpoints (Section 3.1.2. IQWiG GM 4.0).

Validation should be performed within a sufficiently defined patient population (indication) and comparable interventions (pharmaceuticals with similar mode of activity), which drastically limits transferability. Moreover, the correlation of the effects on the surrogate endpoint and true endpoint) should be high and biologically or pharmacologically plausible. Above all, if the causal relationship is not proven, changes in surrogate endpoints do not allow inferences on changes in patient-relevant endpoints [22].

In a recent Rapid Report by IQWiG on the validity of surrogate endpoints in oncology, data from 21 validation studies on breast cancer and colon cancer of different stages (early or advanced, with or without metastases) were examined [23]. Overall survival rate was identified as the only valid patient-relevant endpoint; no valid surrogate endpoint was identified.

Surrogate outcomes of unclear validity may be accepted by IQWiG as outcomes of interventions for extremely serious diseases with high morbidity and mortality, for which there are no treatment alternatives [24]. Surrogate outcomes that have not been fully evaluated may be used based on the concept of surrogate threshold effects [25].

Composite outcomes are generally also not preferred but accepted if clinical trials reporting single outcomes are not available. Composite outcomes are included in the benefit assessment only if all components are patient-relevant endpoints and also reported separately (Section 7.1.5 IQWiG GM 4.0).

In addition to the potential benefit of an intervention, e.g. a decrease in mortality, the evaluation of potential harm is another essential component of the assessment (Section 3.1.3 IQWiG GM 4.0). To evaluate the benefit-harm ratio, adverse events are to be analysed and presented. Relevant adverse effects may partly or fully counterbalance the benefit of the intervention, may be considered especially important by patients, or be associated with serious morbidity, higher mortality, or significant impact on quality of life.

### Statistical analysis and categorisation of outcome

If the available studies are suitable for meta-analysis, results from individual studies should be pooled quantitatively to enhance the validity and certainty of results (Section 7.2.1, 7.2.2 and 7.3.8 IQWiG GM 4.0, and Section 4.2.5.3 Suppl. G-BA VerfO). The overall quality of systematic reviews is assessed using the validated quality indices from Oxman and Guyatt or the AMSTAR tool. Potential heterogeneity of the study results is to be assessed using the I^2^ measure or other statistical tests for heterogeneity [26]. Possible reasons for heterogeneity such as methodical and clinical aspects or effect modifiers should be investigated. Sensitivity analyses should be conducted to assess the robustness of the results if there are differences between studies in terms of methodological aspects regarding the information retrieval and evaluation, e.g. cut-off dates or selection of effect measures (Section 4.2.5.4 Suppl. G-BA VerfO).

The certainty of results for a given study depends on quality of the evidence base and in particular three factors: the type of study design (evidence level), the internal validity, and the size of the observed effect (Section 3.3.2 IQWiG GM 4.0). A classification system is used to indicate the evidence level of the studies included in the benefit assessment (§ 5 par. 6 AM-NutzenV]. Levels of evidence are defined for systematic reviews of RCTs (I a), RCTs (I b), systematic reviews of prospective comparative cohort studies (II a), prospective comparative cohort studies (II b), retrospective comparative studies (III), case series and other non-comparative studies (IV), and other observations, opinions, or reports (V).

Internal validity at the study level is to be assessed in terms of potential bias, i.e. the discrepancy between the conditions of the study (planning, conduct, analysis, and reporting) and the real-world situation (Section 4.2.4 Suppl. G-BA VerfO; Section 7.1.4 IQWiG GM 4.0). The study findings may thus result from the actual therapeutic intervention or rather from influences of other factors or forms of bias such as selection bias, information basis, attrition bias, reporting bias, and publication bias (Section 7.3.11 IQWiG GM 4.0). The potential of bias is to be evaluated and classified as “high” or “low” for randomised studies.

For internal validity at the endpoint level the following aspects are to be considered: blinding of the endpoint collector, adequate implementation of the ITT-principle, and result-independent reporting. The potential of bias is to be evaluated and classified as “high” or “low” for endpoints used in randomised studies (Section 4.2.4 Suppl. G-BA VerfO; Section 7.1.4 IQWiG GM 4.0).

If studies of appropriate quality and precision are not available, it is one of the principle tasks of IQWiG (Section 1.2.5 IQWiG GM 4.0) to describe the evidence base as such and to draw the conclusion that the currently available evidence base does not allow any reliable recommendations. As a consequence, further evidence of the best-available level of quality may be requested by G-BA (§ 5 par. 3 AM-NutzenV].

Efficacy data from clinical studies are never extrapolated qualitatively or quantitatively even if clinical effectiveness data are not available or limited [24]. For example, if there are no long-term data, short-term data are not extrapolated through modeling.

The generalisability, or external validity, should be evaluated by describing whether and to what extent the study results are transferable to local conditions (e.g. patient population, qualification of physicians) (Section 1.2.6 IQWiG GM 4.0). Studies that reflect the routine setting of the German health care system are particularly relevant.

As seen from the perspective of IQWiG, RCTs that combine generalisability and high result certainty are feasible and desirable (Section 1.2.6 IQWiG GM 4.0). In these “pragmatic trials” [27], “real world trials” [28], or “practical trials” [29] there are no defined study visits and no special requirements for the patients, which would go beyond what is necessary for treatment.

Part of the benefit assessment is to evaluate the actual effects of interventions with the highest possible level of result certainty. The certainty that an additional benefit exists is affected by the quality of the evidence provided. The probability of the additional benefit is assessed with respect to the number of patients and the extent of the additional benefit (§ 5 par. 4 AM-NutzenV] and is differentiated by using the categories proof, indication, hint, no proof and no indication (Section 3.1.1 IQWiG GM 4.0). The requirements for the various conclusions on the evidence base are presented in Table 1 (Section 3.1.4 IQWiG GM 4.0).

**Table 1:** **Requirements for the conclusions of early benefit assessment in Germany**

	Requirement		
**Conclusion**	**Number of studies**	**Certainty of results**	**Effect**
Proof	≥ 2	Mostly high	In the same direction
Indication	≥ 2	Mostly moderate	In the same direction
	1	High	Statistically significant
Hint	≥ 2	Mostly low	In the same direction
	1	Moderate	Statistically significant

### Methodological requirements for single technology appraisal in England (NICE)

#### Legal basis, guidance texts, and information sources

##### Legal and guidance texts related to single technology appraisal

The pivotal legal text related to HTA in England is the Directions of the Secretary of State [4], which defines the purpose of the NICE, i.e. to conduct appraisals and develop guidelines. All relevant methodological requirements and procedural aspects regarding HTA are laid down in guidance documents issued by NICE. The methods of technology appraisal are described in the “Guidance for Manufacturers and Sponsors. The Technology Appraisals Process Series No. 5” (GMS TAPS 5) [5] and the “Guide to the methods of technology appraisal” (GMTA) [30]. The methods considered by NICE to be the most appropriate for the purpose of the appraisal are presented for a defined “reference case” (Section 5 GMTA). Further information is found in the “Specification for manufacturer/sponsor submission of evidence STA” (SMSE STA) [31]. The procedure of the single technology appraisal is described in the “Guide to the single technology appraisal (STA) process” [32].

##### Information sources

The appraisal process relies on information and input from numerous sources, including independent assessment groups, manufacturers, healthcare professionals, clinical specialists and patient/carer representatives, who all act as suppliers of evidence, commentary, and/or analysis (Section 1.1.4 GMTA). The best-available data, which are relevant to the question being addressed, should form the basis of the evaluation (Section 2.1.1 GMS TAPS 5).

As part of the “Sponsor’s submission of evidence”, manufacturers/marketing authorisation holders should list all clinical and follow-up studies sponsored by them or known to them, unpublished clinical data (raw data), part-published data (e.g. in abstract form), confidential data, and any evidence from disease registers (Section 3.3.2 to 3.3.8, 3.3.12, and 4.2.1 GMTA). The search strategy used and the selection process, including the rationale for exclusion and exclusion criteria, should be described in detail (Section 5 SMSE STA).

The structure of the submission document follows a question and answer format. The main text, excluding the pages covered by the template, usually should not exceed 100 pages (SMSE STA). No appendix with source information (clinical study reports, etc.) is required. The “decision problem” (Section A of the dossier) is to be submitted by the manufacturer in advance of the full submission [32]. It should be developed from the final scope issued by NICE and state the key parameters that the information in the evidence submission will address, including population, intervention, comparator(s), outcomes, and subgroups to be considered (Section A SMSE STA).

The Evidence Review Group, which is an independent academic group commissioned by NICE to prepare an assessment report for STAs, may perform independent searches to validate the completeness of the evidence submitted. During its critical review, additional analyses may be requested from the manufacturer or sponsor.

Healthcare professionals from professional organisations of the appropriate clinical disciplines provide a professional view of the technology’s effectiveness in the context of routine clinical practice (Section 4.4.2 GMTA). This evidence supplements the information available from published licensing studies, which examine clinical efficacy under controlled conditions.

Individual patients or carers, or groups of patients or carers are requested to give their views, assessments and evaluations during the appraisal (Section 4.3.1 GMTA) as this evidence is considered unique and realistic expert knowledge about the personal impact of a disease and its treatment (Section 4.3.5 GMTA). Patient experience may also identify limitations in the existing research literature.

Moreover, selected clinical specialists and patient experts provide further written evidence and are invited to attend and actively participate in the meeting of the Appraisal Committee (Section 4.5.1 GMTA).

Finally, NICE may sometimes use publications by other HTA agencies (e.g. HTA reports), clinical guidelines, and the European or national public assessment reports (including the SmPC) [24]. Other sources such as web-based study registries are not considered.

#### Clinical study design and methodology

##### Study design features

The key aspects regarding study design (e.g. participant flow) and methods (e.g. statistical analysis) of the included studies are to be described according to the CONSORT [11] checklist (Section 5.3.1 SMSE]. Details on the search strategy used and results of the quality assessment should be provided for both RCTs and non-RCTs, preferably under consideration of guidance issued by the Centre for Review and Dissemination [33].

While data from well-conducted RCTs are generally preferred, the focus of the appraisal is on effectiveness rather than efficacy, and RCTs may thus be considered not appropriate in some situations (Section 2.1.2 GMS TAPS 5; Section 3.3.2 GMTA). More specifically, licensing RCTs may often be too short in duration, restricted to highly defined patient groups, and use comparator therapies that are not routinely used in national health care practice (Section 3.3.3 GMTA).

Ideally, data should be collected from randomised, controlled, prospective studies with a “naturalistic design” and conducted in a routine care setting (Section 2.1.3 GMS TAPS 5). Studies in a natural and country-specific setting (“community effectiveness”), however, are not available in most cases.

If possible, data from RCTs should be supplemented by good-quality data from experimental or observational non-RCTs or other sources (Section 3.3.5 GMTA). Data should be adjusted by using modeling to evaluate important aspects regarding comparative clinical (and cost) effectiveness such as long-term therapeutic outcome.

As part of the scope, NICE defines the patient population (e.g. age, sex, co-morbidities) and, if appropriate, subpopulations of interest, and the treatment setting (inpatient; outpatient) (Section 2.2.3 GMTA). Since one of the aims of the appraisal is to identify those patients most likely to benefit from treatment, subgroup analyses are generally to be carried out. The choice of subgroups (e.g. ‘high-risk’ patients) are to be determined *a priori* and to be supported by a biological rationale, clinical plausibility, social aspects, or other justified factors (Section 5.10 GMTA). The analysis is to be done only for the primary outcome, be complemented by statistical tests of interaction, and be corrected for multiple comparisons (Section 2.8.4 GMS TAPS 5).

The time span to be covered in the appraisal normally equates to the period during which the main differences in therapeutic effects (and use of resources) are expected to be observed; thus a lifetime horizon should be chosen, for example, if between-treatment differences in survival rate are to be reflected (Section 5.1.15 to 5.1.17GMTA). Usually, modeling is required to extrapolate data from the available evidence and to give more suitable estimates for benefit parameters (Section 5.7 GMTA).

##### Comparator and comparison

As part of the scope, which comprises contributions from manufacturers and sponsors and other stakeholders (e.g. patients, carers, clinical experts), NICE determines which comparators are relevant for the appraisal (Section 2.4.1 GMS TAPS 5). The main comparators are typically the interventions most commonly used in the NHS for the patient group of interest (Section 2.5 GMS TAPS 5). If there is another technology that is more cost-effective than the most frequently used technology, this should also be considered as comparator in addition to best standard care (Section 5.1.7 and 6.2.2 GMTA; Section 2.5 GMS TAPS 5). Thus, there will be often more than one relevant comparator. The choice of comparator is not limited to (branded or generic) pharmaceuticals but may also include non-pharmaceutical interventions (e.g. surgical procedures) (Section 2.2.5 GMTA). Finally, the comparator may also be palliative therapy, no therapy, or a non-licensed intervention if it is routinely used in clinical practice (Section 6.2.4 GMTA) [24].

Direct ‘head-to-head’ comparisons of RCTs are clearly preferred, provided the outcome measures and patient groups are relevant (Section 2.2.8, 3.3.2 to 3.3.3, and 5.2.12 GMTA). If licensing studies were controlled with placebo or a comparator not relevant to standard care in England, modeling may be used to indirectly compare study arms from separate studies with a common comparator (Section 2.5 GMS TAPS 5). When performing indirect comparison or mixed treatment comparisons a rationale for identifying and selecting studies should be presented together with an assessment of quality and heterogeneity (Section 5.2.12 to 5.2.18 GMTA; Section 5.7.2 SMSE STA).

##### Clinical endpoints/outcomes

Endpoints that are to be included in the assessment are identified during the scoping. Outcome measures can be either final (clinical) endpoints or intermediate (surrogate) endpoints. During the appraisal of an intervention all the clinical benefits and costs are evaluated “in the broadest sense” (Section 1 GMS TAPS 5). Clinical outcome data should allow evaluation of long-term clinical effectiveness by measuring the impact on morbidity, mortality, and HRQL. Changes in HRQL (e.g. pain relief) should preferably be reported directly from patients, using a standardised instrument such as EQ-5D, if possible. Data on mortality and quality of life should not only be presented separately but also be combined in form of QALYs. In this way, i.e. by calculating utility measures, cost effectiveness can be evaluated (Section 2.6 and 2.12.2 GMS TAPS 5; Section 5.3 GMTA).

In any case, outcome measures should be relevant to patients and/or carers (Section 2.2.8 GMTA). Patients ideally should themselves assess their status of health at each disease stage (Section 2.6 GMS TAPS 5). Treatment compliance may also be measured (Section 5.3.5 SMSE STA). Ease of use of the technology (patient friendliness) is considered to be included in the HRQL [24]. Finally, experience with the use of the technology is considered relevant if experience is known to affect the efficacy or effectiveness, as in surgical procedures for example [24].

Intermediate (surrogate) outcomes may be included in the assessment if either a strong and consistent correlation between the intermediate outcome and final outcome can be shown or if the outcome measured is large, precise and lasting (Section 2.1.4 GMS TAPS 5). For early appraisals of new technologies modeling using data from intermediate outcomes is acceptable when long-term outcome data are not available. Validation may be postponed until epidemiological data have been collected (Section 2.1.5 GMS TAPS 5). Composite endpoints are included in the assessment [24].

Safety data from comparative RCTs, non-comparative RCTs (e.g. post-marketing pharmacovigilance data) and regulatory summaries are considered in the relative effectiveness assessment (Section 5.9 SMSE STA). Adverse events reported on treatment with the new technology as compared with the comparator compound are to be identified using a defined search strategy and subjected to a quality assessment.

### Statistical analysis and categorisation of outcome

To enhance the evidence base regarding clinical effectiveness, manufacturers and sponsors of product are advised to carry out an up-to-date systematic review which includes data from published and unpublished trials in the relevant patient population. In addition, NICE may itself also commission an independent review (Section 2.8.5 GMS TAPS 5).

Systematic reviews should be conducted according to a previously prepared protocol and based on studies with least potential of bias (Section 4.2.3, 5.2.2, 5.2.4 and 6.2.6 GMTA). Eligible studies must be assessed using appropriate inclusion and exclusion criteria (Section 5.2.5 GMTA). Before further data analysis, potential treatment effect modifiers such as patient characteristics (e.g. age, sex, severity of disease), care setting, and year of the study should be identified (Section 5.2.7 GMTA).

Data should be pooled and explored further by meta-analysis if the quality of data is adequate (Section 5.2.8 GMTA). The characteristics and limitations should be described for each study in terms of population, intervention, setting, sample size, and validity (Section 5.2.9 GMTA). The degree of heterogeneity, i.e. any variability in addition to that accounted for by chance, and reasons for it should be investigated as far as possible (Section 5.2.10 GMTA).

The uncertainty, i.e. probability of a different decision, and limitations associated with the analyses of clinical effectiveness should be evaluated using sensitivity analyses (Section 5.2.10 and 5.8.4 GMTA). Potential bias and uncertainty may result from selective use of information, parameter precision, and structural assumptions underlying a model. Therefore, sensitivity analyses should be performed to critically evaluate the effects of incorporating or excluding parts of the information supplied as evidence for the appraisal (e.g. a specific study) (Section 5.2.10, 5.8.4 to 5.8.6 GMTA); probabilistic sensitivity analysis should be performed to characterise uncertainty associated with parameter precision, i.e. distribution around the mean parameter values (Section 5.8.7 5.8.4 GMTA); and sensitivity analyses should be performed to test alternative choices of time span, key parameter, or other methodological aspects (e.g. classification of disease stages; choice of treatment modalities) when using modeling techniques (Section 5.7.1, 5.7.7 and 5.8.5 GMTA).

A classification system is used to indicate the level of evidence of the included studies. The best-quality evidence can be obtained from experimental studies (RCTs) with high internal and external validity (Section 3.3.2 to 3.3.3 GMTA). Lower validity, i.e. greater potential for bias with regard to performance, measurement and attrition, is found in uncontrolled observational studies (Section 3.3.4 to 3.3.5 GMTA). In any case, the value of the available evidence also depends on its relevance for the appraisal to the question defined in the scope.

The uncertainty and limitations associated with the evidence available should be clearly presented (Section 3.2.2 GMTA). To this end, the quality of a study (internal validity) should be assessed on the basis of study design characteristics and aspects related to study conduct (Section 3.3.3 GMTA; Section 5.4.1 SMSE STA). Potential bias, which may lead to a systematic deviation of the effects estimated from study data from the true effect, may be introduced by the method of blinding, method of randomisation, concealment of allocation, duration of follow-up, similarity of groups at baseline, overall size of study population (and imbalances in drop-out rates), selection, measurement, and reporting of outcomes, and data analysis (intention-to-treat analysis; handling of missing data). Potential bias and uncertainty, which may result from the selective use of information from studies and estimates of clinical effectiveness, should be quantified as far as possible (Section 5.2.3 and 5.8.5 to 5.8.6 GMTA).

Rapid evaluations for new interventions are often conducted based on data from licensing studies before long-term clinical data of best quality are available. At the same time, the technology appraisal should reflect the time period during which the main effects on health and use of health care resources are expected or observed (Section 5.1.15 and 5.7.7 GMTA). In this situation, extrapolation (or modeling) is considered appropriate to make best use of the existing data to allow for a thorough assessment (Section 2.7 GMS TAPS 5). Modeling may also be required if the patient population, clinical settings, choice of comparator, type of outcomes are not directly relevant for the appraisal decision (Section 5.7.2 and 5.7.5 GMTA; Section 2.7 GMS TAPS 5). In general, qualitative description of generalisability or quantitative extrapolation (modeling) should be used to transform efficacy data, as far as possible, to effectiveness data, i.e. to adjust data from controlled trials to what would be expected in clinical practice. Currently accepted best practices should be used e.g. [34],[35] and all methodological aspects, including the model structure, any assumptions made, and data sources should be clearly presented and scientifically justified (Section 5.8.2 GMTA).

Concluding the appraisal, NICE classifies its decision or recommendation regarding clinical effectiveness into one of four categories [36]: recommended; optimised (i.e. recommended for a specific subgroup of patients; only in research (e.g. in the context of a clinical trial); not recommended.

## Discussion

The guidelines that describe the methods used for the comparative benefit assessment in Germany and England are generally quite comprehensive. All documents are publicly available; the current IQWiG Methods paper 4.0 is available both in German and English (Table 2).

**Table 2:** **Guidance texts and information sources for early benefit assessment in Germany (G-BA/IQWIG) and single technology appraisal in England (NICE) – a comparison**

Methodological element	Benefit assessment in Germany (G-BA/IQWIG)	Single technology appraisal in England (NICE)
**Guidance on methodology**	Comprehensive, very detailed, and available publicly and partly in English.	Comprehensive, detailed, and publicly available.
**Information sources used**		
Dossier from manufacturer	> 300 pages, plus usually > 10,000 additional pages in confidential Module 5.	< 100 pages (no additional appendix); section on “decision problem” to be submitted in advance.
Providers of other evidence and/or input	Written and oral comments from medical experts, patient organisations, pharmaceutical companies, industry organisations, pharmacists’ associations, and umbrella organisations of medical professionals on IQWiG’s assessment.	Evidence and input from assessment groups, manufacturers, patients, carers, and health care professionals throughout the appraisal.
Studies	All (licensing and other) studies.	All (licensing and other) studies.
Publications	Literature search.	Literature search (& independent search).
Data	Published and confidential.	Published, unpublished, and confidential.
Others	HTA reports (rarely) and web-based study registries.	HTA reports, public assessment reports, and clinical guidelines.

*Abbreviation*: *G-BA* Federal Joint Committee, *HTA* Health technology appraisal, *IQWiG* Institute for Quality and Efficiency in Health Care, *NICE* National Institute for Health and Care Excellence.

The main source of information for the appraisal in both countries is a dossier that is prepared by the manufacturer. The specifications for the dossier to be submitted to G-BA in Germany are very detailed, comprehensive and to be followed strictly. By contrast, the submission of evidence by manufacturers and sponsors for appraisal of single technologies in England is less structured and detailed and allows for some flexibility.

While a few other information sources are used similarly, there is some divergence between the two HTA systems regarding the use of web-based study registers, confidential data, clinical guidelines, and regulatory European or national assessment reports. NICE requests written and oral input from patients, carers, and health care professionals, clinical specialists and patient experts from the start (scoping) up to the Appraisal Committee meeting, which highlights the emphasis on the perspective of these groups. In Germany, medical experts, patients and patient organisations are approached in writing by IQWiG before its 3-month dossier review. Medical experts, patient organisations, pharmaceutical companies, industry organisations, pharmacists’ associations, and umbrella organisations of medical professionals can comment on IQWIG's assessment in writing and orally. Moreover, the Subcommittee Pharmaceuticals of the G-BA, which is responsible for benefit and cost-benefit assessment, also comprises members from patient organisations.

Both HTA institutions consider study design features (study type, (sub)population(s), and duration) to be critical for the level, quality and relevance of the evidence base (Table 3). While similarities prevail, NICE appears to prefer that any kind of additional “soft” evidence be presented if it is expected to be of relevance from the patient/carer perspective. If possible, data should be adjusted or extrapolated via modeling to extract useful information from studies of lower level (e.g. from non-RCTs) or relevance (e.g. from too short studies). It remains to be seen whether G-BA decisions with the condition to conduct additional studies will be the exception or the rule and what kind of additional studies will be required in which situations.

**Table 3:** **Clinical study design and methodology for early benefit assessment in Germany (G-BA/IQWIG) and single technology appraisal in England (NICE) – a comparison**

Methodological element	Benefit assessment in Germany (G-BA/IQWIG)	Single Technology Appraisal in England (NICE)
**Study design features**		
Description	According to international standards.	According to international standards.
Study type	RCTs clearly preferred; case-series acceptable if dramatic therapeutic effect.	RCTs preferred; non-RCTs and other evidence also desired (adjustment of data through modeling).
Subgroup analysis	Always done (effect modifier; interaction tests).	Always done (statistical tests).
Study duration	Important criterion for relevance of evidence (guidelines).	Data extrapolation through modeling.
**Comparator and comparison**		
Choice of comparator	Preferably found beneficial in previous assessments; most economic (if alternatives); preferably reference priced; licensed; can be non-drug intervention; consultation procedure request (advice from G-BA); the comparator is determined by G-BA; often more than one comparator.	Best standard care (most commonly used, most cost-effective, also non-licensed or no intervention); input from manufacturer and other stakeholders during scoping; often more than one comparator.
Direct and indirect comparisons	Direct comparison preferred; indirect comparison possible.	Direct comparison preferred; Indirect comparison possible.
**Endpoints/outcomes**		
Clinical endpoints	Relevance to patients (mortality, morbidity, quality of life); reporting by patients (e.g. HRQL, symptom scores).	Relevance to patients (mortality, morbidity, quality of life) or carers; reporting by patients (e.g. HRQL); translatable into utilities (e.g. QALYs); ease of use of the technology; experience with use of the technology.
Surrogate endpoints	Validation study applicable to the disease, its severity, the intervention, and the comparator required (exception: very serious diseases).	Accepted if correlation with final endpoint is strong or outcome measures are large.
Composite endpoints	Accepted if components patient-relevant and also reported separately.	Accepted.
Safety	Analysis of relevant adverse events.	Analysis of relevant adverse effects.

*Abbreviation*: *G-BA* Federal Joint Committee, *HRQL* Health-related quality of life; *IQWiG* Institute for Quality and Efficiency in Health Care, *ITT* intention-to-treat, *NICE* National Institute for Health and Care Excellence, *QALY* quality-adjusted life year, *RCT* randomised controlled trial.

The decision of G-BA and NICE on the comparator may be revised if the context of health care provision changes and medical progress advances. If a new pharmaceutical shows a significant additional benefit, it may become a future comparator in that indication. Complexity further increases in therapeutic indications like oncology: Different comparators will have to be selected for each specific type of tumour (e.g. breast, lung, prostrate, colon cancer), disease severity (early vs. advanced stage), and treatment modality (mono vs. combination therapy). By contrast, for technology appraisal in England, non-licensed or no interventions are also accepted, and manufacturers have the opportunity to contribute to the choice of comparator during the early scoping process and when submitting the draft “decision problem” in advance of the full submission. In Germany, it is possible and generally advisable for the pharmaceutical companies to seek advice from G-BA on the choice of the appropriate comparator before preparing the dossier or even when planning the pivotal phase 3 studies.

For both G-BA/IQWiG and NICE the preferred outcomes are patient-relevant and related to mortality, morbidity and/or quality of life. For HTA in England, outcomes should, in addition, be translatable into utilities (e.g. QALYs). Ease of use of the technology (i.e. patient friendliness) and experience with the use of the technology (e.g. prescribing rate of the pharmaceutical) are also included as endpoints relevant to the patient and carer perspective.

Surrogate outcomes are generally not favoured and are considered less relevant for the final decision than clinical outcomes. Usually, they are accepted by G-BA/IQWiG only if they have been validated using a meta-analysis (based on several independent RCTs) and a direct causal relationship with the final outcome is proven. It is likely that the cost and time required to fully confirm the scientific validity of a surrogate endpoint exceeds the effort required to conduct a single mega-trial that includes the clinically relevant “hard” endpoint. Nevertheless, according to current guidance in Germany, surrogate outcomes of unclear validity are accepted only exceptionally, in case of extremely serious diseases for which no alternative therapeutic options exist. By contrast, data from intermediate outcomes are acceptable to NICE for early appraisals of new technologies when long-term data are not available. Appropriate modeling should be used and validated when epidemiological data have been collected over time.

As another major difference between the two HTA systems, efficacy data are never extrapolated for benefit assessment in Germany if data on clinical effectiveness are limited or absent (Table 4)*.* In England, however, both qualitative and quantitative extrapolation is performed to adjust data if the study duration, patient population, choice of comparator, or type of outcomes is not directly relevant for the appraisal decision. Modeling techniques are routinely applied to gain information on clinical effectiveness (e.g. if long-term data are absent), as part of the cost-effectiveness analysis, and to evaluate the generalizability of study results (external validity).

**Table 4:** **Statistical analysis and categorisation of outcome for early benefit assessment in Germany (G-BA/IQWIG) and single technology appraisal in England (NICE) – a comparison**

Methodological element	Benefit assessment in Germany (G-BA/IQWIG)	Single Technology Appraisal in England (NICE)
**Systematic reviews & meta-analyses**	Assessment of review quality; assessment of heterogeneity.	Assessment of data quality; assessment of heterogeneity.
		NICE may commission an additional independent review.
**Sensitivity analyses**	Done with regard to methodological aspects of information retrieval and evaluation.	Done with regard to methodological aspects of information retrieval and evaluation, and uncertainty associated with parameter precision (probabilistic sensitivity analysis).
**Quality of evidence base**		
Level of evidence	Clearly defined levels of evidence.	Defined levels of evidence.
Quality of studies (internal validity)	Classification of potential bias.	Quantification of potential bias.
	G-BA may request further evidence as part of the decision.	NICE guidance may be reviewed/up-dated 1–5 years after initial appraisal.
Validity of endpoints	Classification of potential bias	Quantification of potential bias
	(blinding, ITT, reporting).	(blinding, ITT, reporting).
**Extrapolation of results (modeling)**	Not done.	Qualitative extrapolation and quantitative modeling of data regarding study duration, patient population, choice of comparator, and type of outcomes.
**Generalisability of study results (external validity)**	Descriptive evaluation.	Qualitative description or quantitative extrapolation (modeling).
**Categorisation of outcome**	Proof/indication/hint/no proof of (lack of) (additional) benefit (or harm).	Recommended/optimised/only in research/not recommended.

*Abbreviation*: *G-BA* Federal Joint Committee, *IQWiG* Institute for Quality and Efficiency in Health Care, *ITT* Intention-to-treat, *NICE* National Institute for Health and Care Excellence.

Some of the discrepancies in terms of methodological requirements between the two HTA systems may, at least in part, be explained by differences in procedural aspects and differences in the robustness of the data assessed at distinct stages of the technology’s life cycle. The STA process is initiated very early, often several months before marketing authorisation has been granted. Thus, the manufacturer submission of evidence may be received and fully reviewed by the Evidence Review Group even before a CHMP positive opinion is issued [32]. By contrast, the dossier is submitted at market entry in Germany.

At the same time, the different purpose of the assessment process may also explain differences in methodological requirements (e.g. use of modeling). While NICE makes a recommendation regarding reimbursement based on the (combined) appraisal of clinical and cost-effectiveness, G-BA/IQWiG quantify the additional benefit and its results certainty (e.g. proof, indication, or hint of additional benefit). This information will be used, in a second step, to support the price negotiations between the pharmaceutical company and the statutory health insurances (Figure 1).

Finally, in England, any recommendation or guidance issued by NICE will automatically be considered for review or update 1 to 5 years after the initial appraisal. The exact review date depends on the rate at which evidence for the technology is expected to develop, and on the planned reporting of pivotal studies that are underway [32]. In Germany, there is no automatic re-review of the assessment outcome. G-BA may issue a resolution for the initial benefit appraisal with a time restriction, however (§ 1 par. 2 G-BA VerfO). Moreover, at the earliest 1 year after the initial assessment, G-BA may initiate a new benefit assessment because of new scientific findings; likewise, manufacturers may request a re-assessment. In this way, the conclusions reached at the time of initial benefit assessment can be revised, if deemed necessary, when new, relevant evidence emerges.

## Conclusions

The results indicate that there is some degree of similarity regarding basic methodological elements such as selection of information sources, quality assessment of available evidence, choice of clinical endpoints, and choice of comparator(s). In comparison with G-BA/IQWiG, however, NICE appears to generally assume a less restrictive position: Surrogate endpoints may be accepted more readily - provided they are reasonably likely to predict clinical benefit - since full validation may be postponed. Modeling is not acceptable for benefit assessment in Germany but is expected to be performed for HTA in England wherever possible and appropriate, e.g. for study design features such as study duration, patient population, choice of comparator, and type of outcomes. There is also a greater focus on the patient and carer perspective, as evidenced by both methodological elements (outcomes) and process-related aspects (input during scoping and throughout the appraisal). Somewhat greater flexibility is offered by requesting the view from the manufacturer regarding the choice of comparator early on in the process, i.e. during the scoping and preparation of the decision problem.

Overall, the approach taken by NICE seems to make use of best available evidence, including data from non-RCTs. The data are transformed using modeling techniques and the resulting uncertainty is quantified through sensitivity analyses before making a recommendation regarding reimbursement. This contrasts with a more conservative approach taken by G-BA/IQWiG, which bases its assessment of the additional benefit largely, if not exclusively, on evidence of the highest level and quality as well as data on “hard” clinical endpoints. While NICE undertakes a combined appraisal of clinical and cost-effectiveness G-BA/IQWiG does not assess cost-effectiveness at the early stage of benefit assessment. This may explain some of the differences found, e.g. with respect to the use of modeling.

## Authors’ original submitted files for images

Below are the links to the authors’ original submitted files for images. Authors’ original file for figure 1 Authors’ original file for figure 2 Authors’ original file for figure 3
