# Supplementary figures and images for: Requirements for benefit assessment in Germany and England – overview and comparison
Source: Health Econ Rev. 2014 Aug 28;4:12. doi: 10.1186/s13561-014-0012-8 (PMC4884042; doi:10.1186/s13561-014-0012-8)

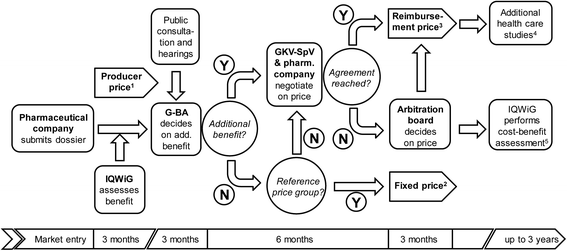

Supplement: Supplementary file 1 — Authors’ original file for figure 1 [file 13561_2014_12_MOESM1_ESM.gif]

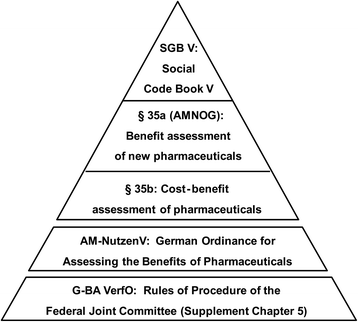

Supplement: Supplementary file 2 — Authors’ original file for figure 2 [file 13561_2014_12_MOESM2_ESM.gif]

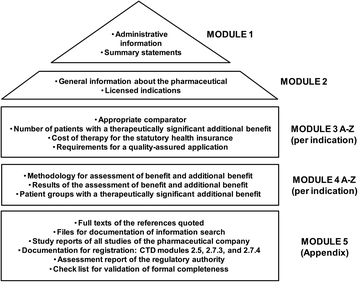

Supplement: Supplementary file 3 — Authors’ original file for figure 3 [file 13561_2014_12_MOESM3_ESM.gif]
